# Tunable Broadband Terahertz Waveband Absorbers Based on Fractal Technology of Graphene Metamaterial

**DOI:** 10.3390/nano11020269

**Published:** 2021-01-20

**Authors:** Tong Xie, Dingbo Chen, Huiping Yang, Yanhong Xu, Zhenrong Zhang, Junbo Yang

**Affiliations:** 1 Guangxi Key Laboratory of Multimedia Communications and Network Technology, School of Computer, Electronics and Information, Guangxi University, Nanning 530004, China; 1913391023@st.gxu.edu.cn (T.X.); 1913391027@st.gxu.edu.cn (Y.X.); 2 Center of Material Science, College of Liberal Arts and Sciences, National University of Defense Technology, Changsha 410073, China; chendingbo15@nudt.edu.cn (D.C.); 201821521341@smail.xtu.edu.cn (H.Y.); 3 School of Physics and Optoelectronics, Xiangtan University, Xiangtan 411105, China

**Keywords:** terahertz, graphene, fractal, broadband

## Abstract

In this paper, a metasurface Terahertz absorber based on the fractal technology of a graphene geometry resonator to realize ultra-wideband, ultrathin, adjustable double-layer cross-fractal formation is introduced. This paper proposes a dynamically tuned graphene absorbing material. The structure is composed of one- to four-level-fractal graphene pattern layers, MgF_2_ layers and metal reflective layers to form a two-sided mirror of an asymmetric Fabry–Perot cavity. To confine the terahertz electromagnetic wave, four different fractals are integrated into a supercell, and the coupling and superposition of adjacent resonant cavities form a broadband high-absorption absorber. Using finite element-based full-wave electromagnetic simulation software to simulate the response frequency of 0.4–2.0 THz, we found that the absorber achieves a broadband 1.26 THz range (absorption > 80%) and a relative bandwidth of 106.8%. By adjusting the Fermi energy, it can realize free switching and expand to wider broadband terahertz absorption, by adjusting the polarization angle (Φ) from 0 to 90° to prove that the structure is not sensitive to polarization, the absorber provides a 60° large angle of incidence, polarization for TE and TM the absorption pattern remains basically the same. Compared with the previous work, our proposed structure uses fractal technology to expand the bandwidth and provide dynamic adjustable characteristics with great degrees of freedom. The appearance of the fractal structure reduces the difficulty of actual processing.

## 1. Introduction

The terahertz (THz) or T-ray region of the electromagnetic spectra is defined as the region between infrared (IR) light and microwave radiation, ranging from 100 GHz to 10 THz. This region lacks natural materials with valuable electromagnetic properties and is extremely precious [1,2]. The technological advancement in the field of terahertz has brought many potential applications, which can be used for medical and security imaging, sensors, biological sensing, wireless communication and intracellular protein composition analysis [3,4,5,6,7,8,9]. Metamaterials are generally periodic structures composed of sub-wavelength micro-nano structural units. This structure breaks through the diffraction limit of traditional optics and produces a series of peculiar optical phenomena including negative refraction. Invisibility cloaks [10], chiral optics [11], gradient metasurfaces [12] and other fields play an important role, and absorbing metamaterials have become an important branch in the application of metamaterials. After Landy [13] and others first proposed a thin and nearly perfect absorbing metamaterial in 2008, metamaterial absorbers began to flourish [14,15,16,17,18]. In many practical application scenarios, such as photoelectric detection, military stealth, radar and communication technology, there is a need for absorbing materials with broadband absorption. Afterwards, researchers turned their attention to dual-frequency, multifrequency and broadband [19,20,21] research on the absorbing metamaterials.

Graphene is the thinnest 2D material found so far. Graphene has the characteristics of small thickness, high elasticity and durability, and low loss. It exhibits metallic properties when certain conditions are met and becomes one of the most promising materials [22,23,24]. Graphene supports surface plasmon polaritons (SPPs) in the middle, far infrared, and the terahertz bands, so graphene-based metasurfaces are expected to be candidates for perfect absorption of terahertz waves. More importantly, compared with the traditional metal metamaterial structure, the surface conductivity of graphene varies with Fermi energy, which can be dynamically adjusted by applying a bias voltage or chemical doping to achieve tunable absorption. The emergence of graphene provides an ideal material that can replace metal structures [25,26,27].

Fractal theory is closely related to many research fields with a wide range of applications, and the fractal phenomenon of self-similarity will exist. Traditionally, all cells of the fractal have the same geometric shape, the same size, and the same arrangement direction, which are derived from the space filling of the fractal structure and are on the same plane, which reduces the difficulty of processing [28,29]. However, fractal is a kind of similar shape between its components and the whole. It can be said to be a tangible fractal structure in the traditional sense or a similar intangible fractal structure in a statistical sense [30]. The fractal metamaterial technology that uses fractal structure to realize the characteristics of metamaterials is an emerging technology. The fractal structure can be used to construct cells with metamaterial properties. Compared with traditional metamaterial cells, fractal metamaterials also have the advantages of miniaturization, ultra-wideband, high absorption, and multiple resonances. Based on the frequency shift of different fractal series and multiple resonant frequency bands, by integrating different fractal resonators into a supercell, the coupling and superposition of adjacent resonators obtains the desired ultra-wideband response [31,32,33]. Although from the perspective of absorption characteristics, the existing fractal terahertz absorbers based on metal metamaterials have excellent absorption characteristics, due to the limitations of the fixed characteristics of the commonly used metal metamaterials, they will absorb the function of the wave device, which is single, does not have the flexibility of tunability, and cannot adapt to various complex application scenarios. Based on this, we numerically demonstrate an ultra-wideband, ultrathin, and adjustable metasurface terahertz absorber using the concept of fractal supercells.

In this work, the structure we designed is composed of a one- to four-level fractal graphene pattern layer, MgF_2_ layer and metal reflective layer. Based on this structure, we mainly integrate two layers of four different fractal structures into a supercell, so a broadband high-absorption absorber is formed. Therefore, our device provides dynamic adjustable characteristics with a great degree of freedom, and our proposed absorber can realize an ultra-wideband, ultrathin, and adjustable planar metasurface terahertz absorber. Using simulation software to simulate the response frequency of 0.4–2.0 THz, we found that the absorber achieves a broadband 1.26 THz range (absorption > 80%), where the center frequency *f*_c_ *=* 1.18 THz and the relative bandwidth is 106.8%, achieving broadband absorption by optimizing each fractal structure. In addition, each level of fractal structure we design presents a periodic symmetry pattern, the polarization angle (Φ) is tuned from 0 to 90°, which means that the structure isn’t sensitive to the polarization angles. The oblique angle can be incident at a large angle up to 60° for both TE and TM polarizations is achieved.Compared with the previously reported fractal terahertz metamaterial absorbing materials, the structure we designed has the advantages of easy realization, low cost, miniaturized manufacturing and adjustable degree of freedom, which is useful for future terahertz detectors, terahertz communications and other emerging the realization of terahertz technology is particularly important.

## 2. Materials and Methods

**Figure 1:**
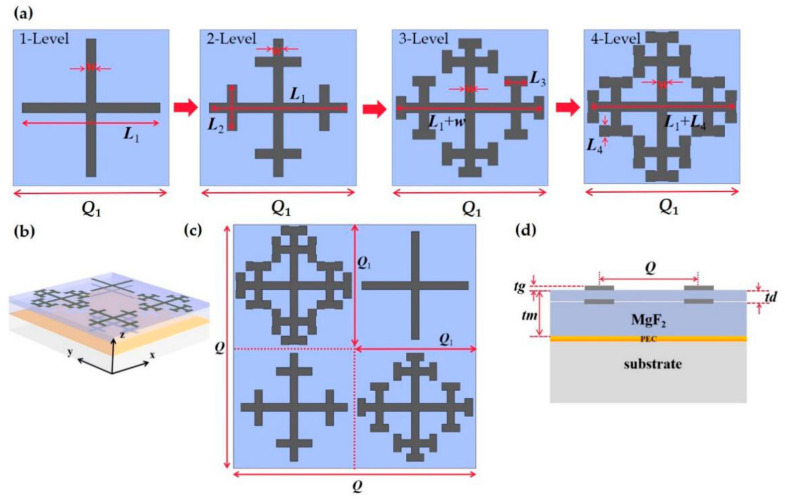
Fractal supercell absorber. (**a**) The evolution of the cross fractal from one to four-level fractals. The three-level and four-level fractals are optimized structures. (**b**) Structure diagram of a tunable broadband terahertz absorber based on fractal technology graphene. (**c**) Supercell synthesized by one- to four-level fractals. (**d**) Side of supercell absorber.

The metamaterial absorber we propose is composed of a fractal graphene pattern layer, an MgF_2_ layer and an ideal electrical conductor (equivalent to the ground plane of the metal reflective layer), as shown in Figure 1b,d. As the dielectric layer, the selected dielectric spacer material is MgF_2_, the relative dielectric constant *ε_d_ =* 1.9 [34] and the thickness of *t_m_* = 45 μm. The structure we designed underwent resonant increased cross by four-level fractals. As shown in Figure 1a, starting from the one-level fractal, each level of fractal is based on the original basis with a proportional increase of a self-similar shape, this is equivalent to increasing the effective length to the original size, and then arranging the positions according to the simulation optimization. The resonance frequency also shows a red shift with the increase of the fractal series [33,35]. In order to ensure that the width setting does not affect the four-level fractal, the width of all resonator lines is set to 3.6 μm, which is also to ensure the beauty of the grading four-level fractal. That is, *L*_1_ = 45 μm, *L*_2_ = 15 μm, *L*_3_ = 7.5 μm, *L*_4_ = 3.75 μm, *w* = 3.6 μm. The length of the three-level fractal is slightly increased to 48.6 μm, which is *L*_1_ + *w*, and the length of the four-level fractal is 48.75 μm, which is *L*_1_ + *L*_4_. For our design, the Fermi energy of the top layer graphene corresponds to 1 eV.

In the bottom layer, the size and arrangement of the cross shape embedded in the MgF_2_ dielectric layer are consistent with the first layer, as shown in Figure 1b. After repeated experiments, in order to obtain the best performance, the embedded graphene cross fractal is placed below the top layer (MgF_2_ dielectric layer/air interface) at a distance of *t_d_* = 12 μm, that is, the distance from the ground is 33 μm in Figure 1d. The Fermi energy of the bottom layer is the same as that of the top layer, which is 1 eV. There are two reasons for the same size, arrangement and size of the two layers: first, the top layer has the best absorption rate, and the bottom layer is still the best absorption after being embedded in the dielectric layer when the Fermi energy is 1 eV; second, the same size can reduce the complexity of manufacturing and simulation and reduce process difficulties. Because it is arranged periodically, no matter where the fractal is placed, it has no great effect. Considering the complexity of the three-level and four-level fractal design, it is placed in a diagonal position, that is, a clockwise four-one-three-two level fractal arrangement, as shown in Figure 1c. Since graphene is transparent, in order to obtain a high absorption rate based on the mutual effect of adjacent cells, we optimized the structure to obtain a period of *Q*_1_ = 50 μm. The supercell period *Q* = 100 μm, and the graphene thickness is *t_g_* = 0.5 nm.

The conductivity of graphene is provided by the Kubo equation, and the Kubo equation determines the intraband and interband transitions by both [36,37]:

(1) $$\sigma_{g}\left(\omega ,\mu_{c},\tau ,T\right)=\sigma_{intra}+\sigma_{inter}$$

(2) $$\sigma_{intra}=\frac{2k_{B}Te^{2}}{\pi \hbar^{2}}\times In(2\cos h\frac{E_{f}}{2k_{B}T})\times \frac{i}{\omega +i\tau^{-1}}$$

(3) $$\sigma_{inter}=\frac{e^{2}}{4\hbar }\times \left[H(\frac{\omega }{2})+i\times \frac{4\omega }{\pi }\times \int_{0}^{\infty }\frac{H(\Omega )-H(\frac{\omega }{2})}{\omega^{2}-4\Omega^{2}}d\Omega \right]$$

Here, $H\left(\Omega \right)=\sin h\left(\frac{\hbar \Omega }{k_{B}T}\right)/\left[\cos\left(\frac{\hbar \Omega }{k_{B}T}\right)+\cos h\left(\frac{E_{f}}{k_{B}T}\right)\right]$, and *k*_B_ is Boltzmann’s constant, ℏ=h/2π is the reduced Planck’s constant, *h* is the Planck’s constant, *T* is the Kelvin temperature, *ω* is frequency of the electromagnetic wave, *e* is the elementary charge,$\mu_{c}=10^{4}{\mathrm{cm}}^{2}/V$, the Fermi energy of graphene $E_{f}$ and Fermi velocity $\upsilon_{f}=10^{6}m/s$, $\tau =\mu E_{f}/e\upsilon_{f}^{2}$ is the carrier relaxation time.

In the terahertz band, the conductivity of graphene is mainly provided by the intra-band transport of carriers, while the inter-band transport is suppressed. At this time, the above calculation formula for graphene conductivity is simplified as:

(4) $$\sigma_{g}\left(\omega \right)=\frac{e^{2}E_{f}}{\pi \hbar^{2}}\times \frac{i}{\omega +i\tau^{-1}}$$

In addition, electrical conductivity will directly affect the characteristics of surface plasmon polariton (SPP) excitation.

It can be seen from formula Equation (5) that the conductivity affects the dielectric constant of the graphene film. Graphene is an ultra-thin film with a certain equivalent dielectric constant:

(5) $$\varepsilon_{g}\left(\omega \right)=1+\frac{i\sigma_{g}\left(\omega \right)}{\varepsilon_{ο}\omega t_{g}}$$

Therefore, it can be flexibly manipulated using applied voltage, temperature, carrier concentration, etc., which can be obtained by creating subwavelength composite materials, where *ε_o_* is the dielectric constant of the vacuum and *t_g_ =* 0.5 nm is the thickness of the graphene. The wavelength of electromagnetic waves is much greater than the thickness of graphene, which is a boundary condition that can be used in transition simulation.

The Fermi energy of graphene mainly depends on the carrier concentration *n*_s_, by the equation:

(6) $$E_{f}=\hbar \upsilon_{f}\times \sqrt{\pi n_{s}}$$

Here *n*_s_ is the carrier concentration. Bias voltage and chemical doping can change the carrier concentration, but it is proportional to the bias voltage applied to the graphene layer and *ε_d_* is the relative permittivity of the dielectric layer, *H* is the thickness of the dielectric layer. Equation (7) gives a specific conversion relationship:

(7) $$n_{s}=\frac{\varepsilon_{d}\varepsilon_{o}}{eH}\times \left|V_{biased}\right|$$

The above theory fully demonstrates that the absorbing material applies bias voltage or chemical doping to achieve dynamic adjustment, so as to achieve the purpose of tunable absorption. This process qualitatively changes the frequency of surface plasmon polariton (SPP) excitation characteristics, thereby affecting the absorption the operating frequency of the wave material.

In order to evaluate the function of the double-layer graphene terahertz absorber we designed, we performed numerical simulations using the electromagnetic simulation software based on the finite element method. In the simulation, a cell in the periodic structure is taken as the calculation object, the boundary conditions of the periodic cell in the X and Y directions are selected, and the Z direction is set as the open boundary condition.

The absorptance A(*w*) formula can be written as:

(8) A (ω)= 1−T(ω)−R(ω)

where T(*ω*) and R(*ω*) are the transmission coefficient and reflection coefficient, respectively, and the absorptivity is obtained by using simulation software, selecting the frequency domain solver, and deriving the absorptivity and ***S*** parameters. It can be seen from Equation (8) that, by reducing the transmission and reflection coefficients, higher absorption spectra can be obtained. The ideal electrical conductor in the simulation software is used as a thin metal film reflective layer ground plane to minimize the transmission coefficient. The result shows that the transmission coefficient of the metal layer is zero, A (*ω*) = 1 − R(*ω*).

## 3. Results and Discussions

### 3.1. Narrow Band Absorption of Top and Bottom Two Levels of Fractal

**Figure 2:**
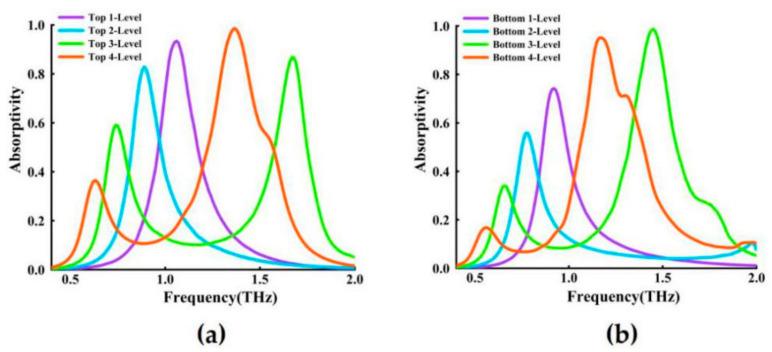
One-level to four-level cross fractal narrow-band absorption spectra: (**a**) top, (**b**) bottom.

Using the parameters of the structure diagram shown in Figure 1, a single cross-shaped fractal array is simulated to obtain its corresponding resonance frequency, Figure 2a shows the top levels of the fractal spectra and Figure 2b shows the bottom levels of the fractal spectra. It can be seen from Figure 2a that with the increase of the fractal order, the resonant frequency of the first peak of the fractal has a significant red shift [33,35]. It can be seen from Figure 2a that the first maximum absorption peaks of the cross one- to four-level fractals are respectively located at: 1.05 THz, 0.89 THz, 0.74 THz, 0.63 THz, but it is worth noting that the three-level fractal (green curve) has a second formant at 1.68 THz, and the four-level fractal (red curve) has at 1.37 THz a second resonance peak.

The bottom layer is composed of graphene fractal structures with the same size, the same arrangement, and the same Fermi energy. Figure 2b shows the resonance absorption spectra obtained by the bottom one- to four-level cross fractals. Figure 2a,b roughly shows a relatively similar trend, but they are not consistent in resonance frequency. It can be seen from Figure 2b that the first maximum absorption peaks of the cross one- to four-level fractals appear at 0.92 THz, 0.77 THz, 0.66 THz, 0.55 THz, and similarly, for the three-level fractal (green curve) and the four-level fractal (red curve), there are second resonance peaks at 1.45 THz and 1.17 THz respectively. It can be seen from Figure 1b that the graphene cross fractal is embedded in the MgF_2_ dielectric layer rather than at the boundary, and the reflection-enhancing response has a small effect on the cross fractal. According to the analysis of Figure 2a,b, theoretically inferred, the broadband range (absorption > 60%) of the double-layer absorber we designed is from 0.5 THz to 1.8 THz.

### 3.2. Broadband Absorption

**Figure 3:**
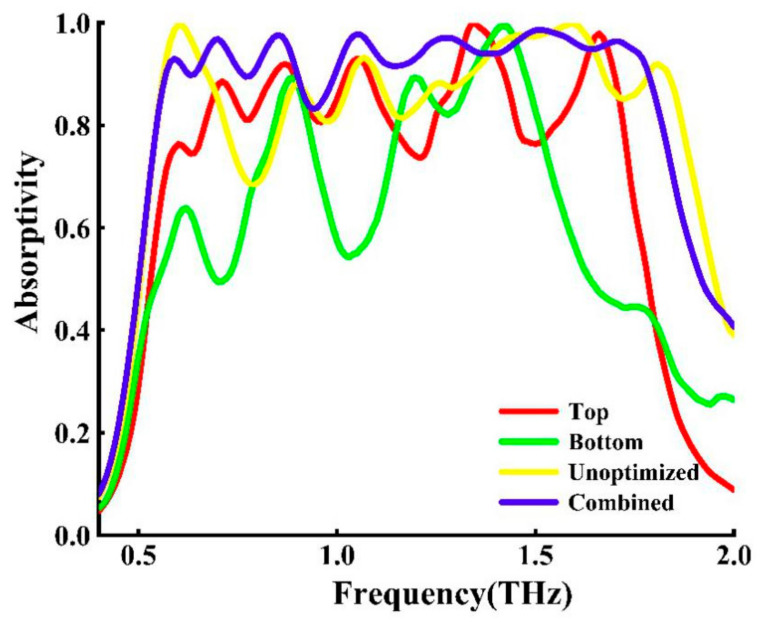
The unoptimized structure and the optimized absorption spectra of the top, bottom and combined layers after optimization. The yellow curve shows the broadband response before the structure is optimized, the red curve shows the broadband response of only the top layer, and the green curve shows the broadband response of only the bottom layer. Using the two-layer structure at the same time, a combined broadband response is obtained, as shown in the purple curve.

**Figure 4:**
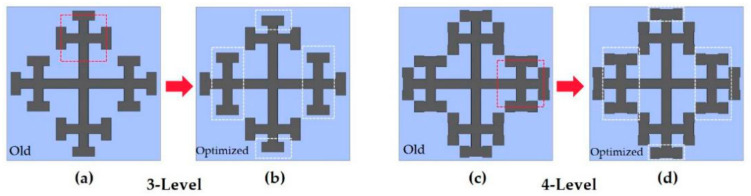
Three-level and four-level fractals, self-similar structures and optimized structures are placed in equal proportions. (**a**) The old three-level fractal structure. (**b**) The optimized three-level fractal structure. (**c**) The old four-level fractal structure. (**d**) The optimized four-level fractal structure.

Through the research of cross fractal absorbers at all levels, we have learned the basic principles of suppressing reflections. However, the narrow absorption bandwidth severely inhibits the application of the absorber, so expanding the bandwidth has become the focus of our work. In the next step, we combine one- to four-level fractals of cross fractals into the supercell structure in Figure 1c to achieve broadband absorption, and show the not optimized structure and the optimized top, bottom, and combined layer absorption spectra the results are shown in Figure 3. As we all know, a fractal is a kind of similar shape between its components and the whole. It can be said to be a tangible fractal structure in the traditional sense or a similar intangible fractal structure in a statistical sense [30]. In the initial stage, we designed the structure to start from a one-level fractal and a two-level fractal is placed on itself when reduced by one third under a one-level fractal. Similarly, three-level fractal is reduced by one sixth under the one-level fractal. It is placed on itself and the four-level fractal can be deduced by analogy. That is, each level of fractal adds a self-similar and proportionally arranged shape on the original basis, which is equivalent to increasing the effective length from the original size. The unoptimized cross fractal structure is shown in Figure 3 (yellow curve). From the absorption curve, it can be seen that the bandwidth and high absorption cannot be formed in the multifrequency range of 0.72–0.85 THz, 1.1–1.33 THz. After that, the data structure was optimized by multiple simulations, and the position of the cross three-level fractal and four-level fractal was adjusted under the condition of the same size, as shown in Figure 4a, which is the old three-level fractal optimization. Figure 4b is the optimized three-level fractal. The top and bottom positions of the three-level fractal are placed at the ratio of *L*/2.17, and the left and right positions are placed at the ratio of *L*/5. The adjustment part is shown as the white dotted line in Figure 4b. Figure 4c is the four-level fractal before optimization, and Figure 4d is the four-level fractal after optimization. The top and bottom positions of the four-level fractal are placed at the ratio of *L*/1.96, and the left and right positions are placed at the ratio of *L/*5.6. The adjustment part is shown as the white dotted line in Figure 4d.

The optimized structure diagram is shown in Figure 1a, and the absorption simulation result is shown in Figure 3 (purple curve). It can be seen from Figure 3 that the top layer cross fractal (red curve) after combination has higher absorption spectra in the range of 0.55–1.76 TH, which is more than 60% consistent with the combination layer, and, as is discussed in Figure 2a,b, the crossover frequency of each graded cross-shaped resonance frequency (0.5–1.8 THz) is almost the same. We found that the absorber achieves (absorption > 80%) in the range of 0.55–1.81 THz. We calculated (FWHM) ∆*f* to be 1.26 THz, where the center frequency *f*_c_ = 1.18 THz, using ∆*f*/*f*_c_ to calculate the relative bandwidth 106.8%.

We attribute the bandwidth and high absorption changes formed by the combination of various fractals to the cross fractal coupling that affects the resonance frequency, which is better than the bandwidth and absorption of the fractal alone. In the same way, the bottom cross fractal in Figure 3 (green curve) also shows a continuous high broadband absorption exceeding 70% in the 0.8–0.95 THz and 1.14–1.64 THz segments. Although the top and bottom cross fractals have the same size and position arrangement, due to the influence of the dielectric layer MgF_2_, the absorption rate is relatively low in some wavebands, and the absorption resonance will also be inconsistent. This frequency range is almost the same as the resonant frequency of each graded cross shape discussed in Figure 2b, but there will be some deviations due to the cross-shaped coupling, but this is not an influencing factor.

### 3.3. E-Fields of Broadband Absorption in the Top and Bottom Layers

**Figure 5:**
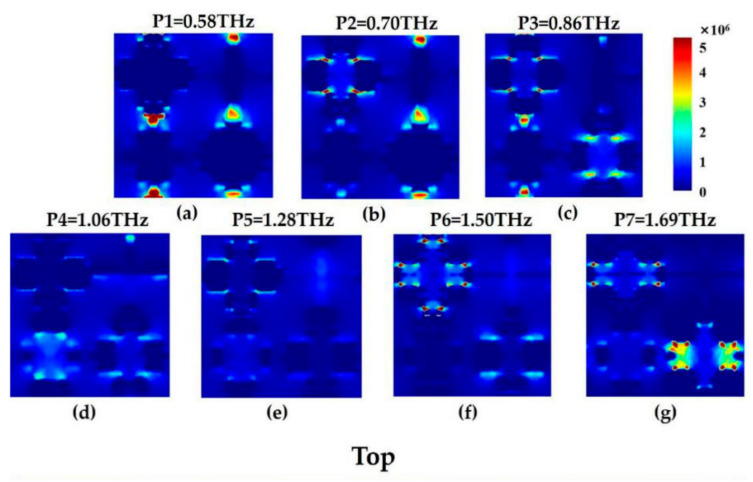

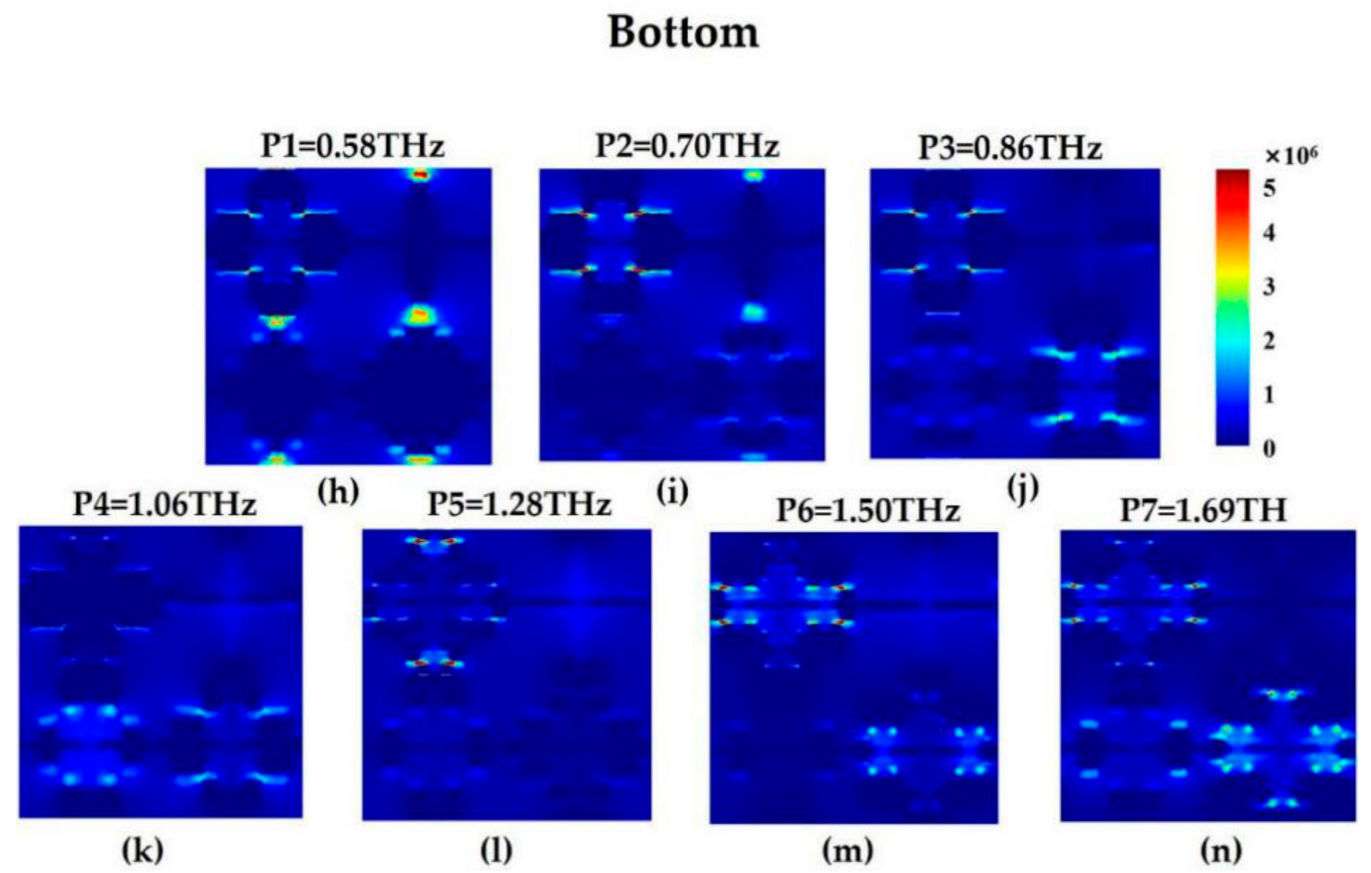
The cross fractals of the top (**a**–**g**) and bottom (**h**–**n**) layers of the absorption spectra correspond to the E- field profiles for broadband peaks.

In order to better study the broadband absorption of the absorber, we use numerical analysis to further discuss the 2D electric field distribution of the resonance absorption spectra of seven high-frequency E-field distributed in two layers within a certain terahertz range, and the selected frequencies are respectively 0.58, 0.70, 0.86, 1.06, 1.28, 1.50 and 1.69 THz. Figure 5a–g corresponds to the seven resonant absorption spectra of the top layer cross-shaped two-dimensional E-field, and Figure 5h–n corresponds to the seven bottom layer resonant absorption spectra of the cross-shaped two-dimensional E-field. In order to ensure the consistency of data comparison, all electric field distributions are on the same standardized scale. It can be seen from Figure 2a,b that there are two resonance peaks in the three-level and four-level fractal. The P1 peak (0.58 THz) is mainly caused by the first resonance peak of the top and bottom three- and four-level fractals. For the P2 peak (0.70 THz), P3 peak (0.86 THz), P4 peak (1.06 THz), the electrical response is more complicated, and it is affected by the top and bottom layers and multiple fractals, or by the coupling and superposition of adjacent resonance peaks. No single layer or single fractal plays a leading role. For the P5 peak (1.28 THz) resonance is only caused by the action of the top and bottom four-level fractals, and the electric field intensity is higher than the other three levels. For the P6 peak (1.50 THz) and P7 peak (1.69 THz), the resonance is mainly caused by the three-level and four-level fractal effects, and the one-level and two-level fractals have no obvious effect on the P6 and P7 peak. To sum up, it has strong resonance in the 0.4–2 THz terahertz band is mainly caused by the interaction, coupling and superposition between the fractals.

### 3.4. Fermi Energy

**Figure 6:**
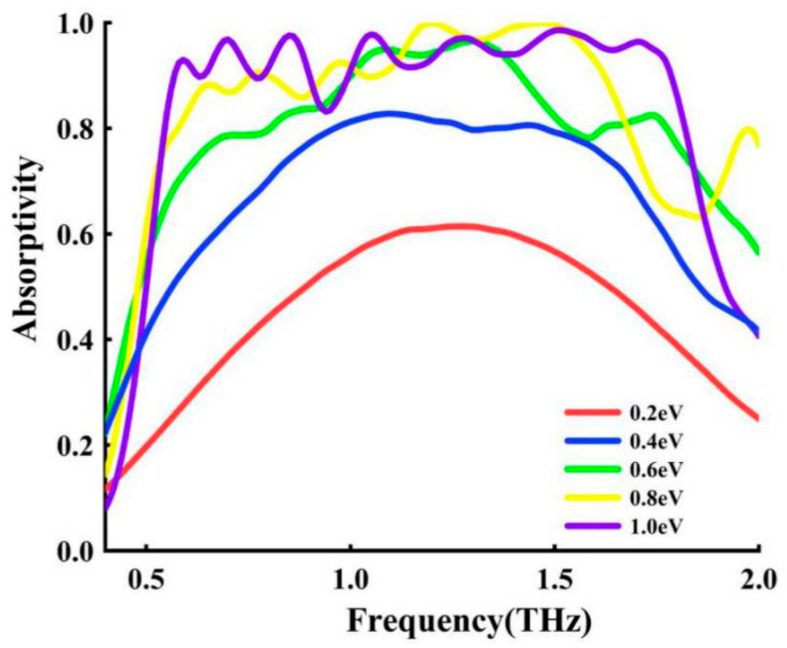
The absorption spectra when the Fermi energy of graphene is 0.2–1 eV under certain other parameters.

After the structure is fixed, the properties of the metal are also fixed, and the appearance of graphene has changed the status quo. As a controllable material, graphene can be dynamically adjusted by applying a bias voltage or chemical doping. It can be adjusted as a whole or locally. It achieves dynamic adjustment of the working frequency and intensity of narrowband and broadband absorption, making the metamaterial there is more freedom [19]. Figure 6 shows the simulated absorption spectra at different Fermi energy. With the adjustment of the Fermi energy, the bandwidth and absorption efficiency are significantly improved from 0.2 eV to 1 eV.

### 3.5. The Thickness of Dielectric Layer

**Figure 7:**
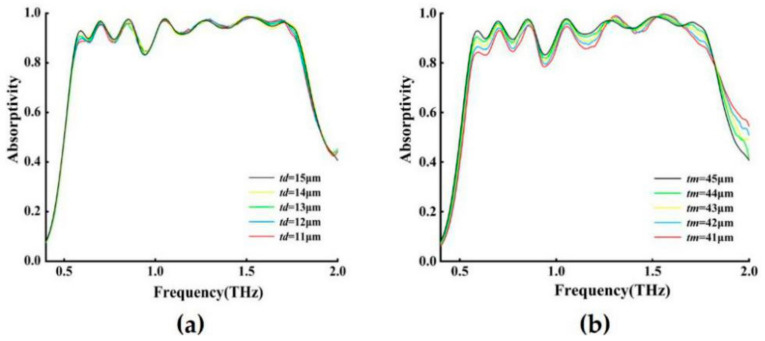
Absorbance of structures with different thicknesses. (**a**) Single-layer dielectric when *Q* = 100 µm, and *t_m_* = 45 µm (**b**) Double-layer dielectric thickness when *Q* = 100 µm, and *t_d_* = 12 µm.

In order to better test the performance of our designed absorber, the influence of the absorption spectra of the top and bottom (*t_d_*, *t_m_*) MgF_2_ dielectric layers of the structure with different thicknesses was studied, and the results are shown in Figure 7. The simulation experiment shows that the best absorption performance can be obtained when *t_m_* = 45 μm and *t_d_* = 12 μm. It can be seen from Figure 7a that *t_d_* does not change significantly between 11–15 μm, but at 0.6–0.9 THz, *t_d_* = 12 μm has a good absorption effect, and the bandwidth is relatively large when *t_d_* = 12 μm, and we can see *t_d_* = 12 μm has the best absorption effect. Figure 7b shows that with the increase of the dielectric *t_m_* layer, the absorption becomes better and better. Under certain other conditions, when *t_m_* = 45 μm, a better absorption bandwidth and high absorption rate can be obtained.

### 3.6. Polarization and Incidence Angle Dependence

**Figure 8:**
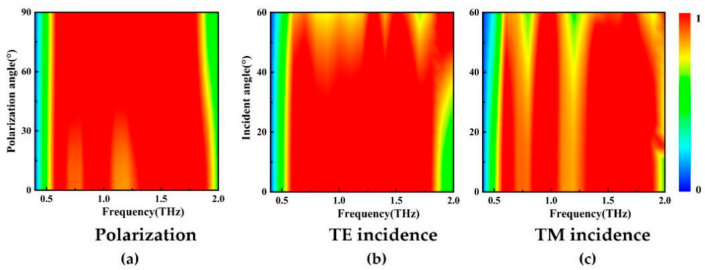
Numerical simulation of the polarization and oblique incidence of the supercell absorber. (**a**)Absorption spectra with different polarization angles (Φ = 0–90°), the polarization angles range from TE polarization (0°) to TM polarization (90°). (**b**,**c**) 0 to 60° oblique incident angle for TE incidence and TM incidence.

In addition to analyzing the influence of the electric field and the dielectric layer, a good standard for measuring the absorber is that its polarization and incident angle are not sensitive. Therefore, we used the simulation software to perform a numerical simulation to check our absorbing structure at different polarizations and oblique incidence angles. Figure 8a is a numerical simulation of the supercell structure of the absorption spectra at different polarization angles (Φ). It can be seen from Figure 8a that the absorption of the tuning polarization angles (Φ) from 0 to 90° remains unchanged for the TE and TM modes. The results show that, because our absorbing structure is periodic symmetric, under normal incident conditions, the absorption is completely independent of polarization, which means that it is insensitive to the polarization angle. In practical applications, the incident light is usually irradiated at an oblique incident angle. We simulate the oblique incident angle from 0 to 60°, and the absorption modes of TE and TM polarization are basically consistent. Figure 8b shows TE polarization, the E-field is always perpendicular to the incident plane. Figure 8c shows TM polarization, and its magnetic field is always perpendicular to the incident surface. The results show that our structure is a highly optimized TE and TM polarized wave incident at a large angle, with good absorption performance and stable working bandwidth.

## 4. Conclusions

In summary, we propose a metasurface terahertz absorber based on fractal technology graphene geometric resonator to achieve ultra-wideband, ultrathin, adjustable double-layer cross fractal formation. We designed the device to achieve (absorption > 80%) in the range of 0.55 THz to 1.81 THz, where the center frequency *f*_c_ = 1.18 THz, and the relative bandwidth 106.8%. Studies have shown that considering the dielectric thickness and electric field strength, there is a higher absorption efficiency, which proves that the absorber we designed is insensitive to polarization angles and large angles of 0 to 60°. According to the bias voltage and chemical doping changes the characteristics of the Fermi energy of graphene, it effectively controls the absorption strength and resonance frequency of the metamaterial absorber, and realizes the dynamic tuning of the metamaterial absorber, which has obvious advantages compared with the traditional metal metamaterial structure. This has important application prospects in optical communication, transformation optics and optical imaging.

## Data Availability

Data is available in the main text.

## References

[1] He Y., Wu Q., Yan S. (2019). Multi-Band Terahertz Absorber at 0.1–1 THz Frequency Based On Ultra-Thin Metamaterial. Plasmonics.

[2] Low T., Avouris P. (2014). Graphene Plasmonics for Terahertz to Mid-Infrared Applications. ACS Nano.

[3] Liu C., Qi L., Wu M. (2018). Triple-Broadband Infrared Metamaterial Absorber with Polarization-Independent and Wide-Angle Absorption. Opt. Mater. Express.

[4] Zhang Z., Yu Y., Fu S. (2017). Broadband On-Chip Mode-Division Multiplexer Based on Adiabatic Couplers and Symmetric Y-Junction. IEEE Photonics J..

[5] Zhou Q., Liu P., Bian L.A., Xin C., Liu H. (2018). Multi-Band Terahertz Absorber Exploiting Graphene Metamaterial. Opt. Mater. Express.

[6] Barzegar-Parizi S. (2018). Realization of Wide-Angle and Wideband Absorber Using Metallic and Graphene-Based Metasurface for Mid-Infrared and Low THz Frequency. Opt. Quant. Electron..

[7] Zhu Y., Hu X., Fu Y., Yang H., Gong Q. (2013). Ultralow-Power and Ultrafast All-Optical Tunable Plasmon-Induced Transparency in Metamaterials at Optical Communication Range. Sci Rep..

[8] Zhang Z., Li J., Wang Y., Qin Y. (2020). Direct Detection of Pilot Carrier-Assisted DMT signals with Pre-Phase Compensation and Imaginary Noise Suppression. J. Lightwave Technol..

[9] Yoshida H., Ogawa Y., Kawai Y., Hayashi S., Hayashi A., Otani C., Kato E., Miyamaru F., Kawase K. (2007). Terahertz Sensing Method for Protein Detection Using a Thin Metallic Mesh. Appl. Phys. Lett..

[10] Ergin T., Stenger N., Brenner P., Pendry J.B., Wegener M. (2010). Three-Dimensional Invisibility Cloak at Optical Wavelengths. Science.

[11] Khorasaninejad M., Chen W.T., Zhu A.Y., Oh J., Devlin R.C., Rousso D., Capasso F. (2016). Multispectral Chiral Imaging with a Metalens. Nano Lett..

[12] Sun S., He Q., Xiao S., Xu Q., Li X., Zhou L. (2012). Gradient-Index Meta-Surfaces as a Bridge Linking Propagating Waves and Surface Waves. Nat. Mater..

[13] Landy N.I., Sajuyigbe S., Mock J.J., Smith D.R., Padilla W.J. (2008). Perfect Metamaterial Absorber. Phys. Rev. Lett..

[14] Song Z., Wang Z., Wei M. (2018). Broadband Tunable Absorber for Terahertz Waves Based On Isotropic Silicon Metasurfaces. Mater. Lett..

[15] Wang T., Zhang Y., Zhang H., Cao M. (2019). Dual-Controlled Switchable Broadband Terahertz Absorber Based On Graphene-Vanadium Dioxide Metamaterial. Opt. Mater. Express.

[16] Song Z., Deng Y., Zhou Y., Liu Z. (2019). Terahertz Toroidal Metamaterial with Tunable Properties. Opt. Express.

[17] Liu H., Wang Z.-H., Li L., Fan Y.-X., Tao Z.-Y. (2019). Vanadium Dioxide-Assisted Broadband Tunable Terahertz Metamaterial Absorber. Sci. Rep..

[18] Xu Z., Gao R., Ding C., Wu L., Zhang Y., Xu D., Yao J. (2015). Photoexited Switchable Metamaterial Absorber at Terahertz Frequencies. Opt. Commun..

[19] Tao H., Landy N.I., Bingham C.M., Zhang X., Averitt R.D., Padilla W.J. (2008). A Metamaterial Absorber for the Terahertz Regime: Design, Fabrication and Characterization. Opt. Express.

[20] Yao G., Ling F., Yue J., Luo C., Ji J., Yao J. (2016). Dual-Band Tunable Perfect Metamaterial Absorber in the THz Range. Opt. Express.

[21] Shen X., Yang Y., Zang Y., Gu J., Han J., Zhang W., Cui T.J. (2012). Triple-Band Terahertz Metamaterial Absorber: Design, Experiment, and Physical Interpretation. Appl. Phys. Lett..

[22] Novoselov K.S., Fal’Ko V.I., Colombo L., Gellert P.R., Kim K.A. (2012). A Roadmap for Graphene. Nature.

[23] Li Q., Tian Z., Zhang X., Singh R., Du L., Gu J., Han J., Zhang W. (2015). Active Graphene–Silicon Hybrid Diode for Terahertz Waves. Nat. Commun..

[24] Shen H., Liu F., Liu C., Zeng D., Meng H. (2020). A Polarization-Insensitive and Wide-Angle Terahertz Absorber with Ring-Porous Patterned Graphene Metasurface. Nanomaterials.

[25] Shi X., Han D., Dai Y., Yu Z., Sun Y., Chen H., Liu X., Zi J. (2013). Plasmonic Analog of Electromagnetically Induced Transparency in Nanostructure Graphene. Opt. Express.

[26] D’Aloia A.G., D’Amore M., Sarto M.S. (2020). Low-Terahertz Transparent Graphene-Based Absorber. Nanomaterials.

[27] Liu Z., Guo L., Zhang Q. (2019). A Simple and Efficient Method for Designing Broadband Terahertz Absorber Based on Singular Graphene Metasurface. Nanomaterials.

[28] Sederberg S., Elezzabi A.Y. (2011). Sierpiński fractal plasmonic antenna: A fractal abstraction of the plasmonic bowtie antenna. Opt. Express.

[29] Moeini S., Cui T.J. (2019). Fractal Coding Metamaterials. Ann. Phys..

[30] Mandelbrot B.B. (1982). The Fractal Geometry of Nature. Am. J. Phys..

[31] Kenney M., Grant J., Cumming D.R.S. (2019). Alignment-Insensitive Bilayer THz Metasurface Absorbers Exceeding 100% Bandwidth. Opt. Express.

[32] Zubair A., Zubair M., Danner A., Mehmood M.Q. (2020). Engineering Multimodal Spectrum of Cayley Tree Fractal meta- resonator Supercells for Ultrabroadband Terahertz Light Absorption. Nanophotonics.

[33] Kenney M., Grant J., Shah Y.D., Escorcia-Carranza I., Humphreys M., Cumming D.R.S. (2017). Octave-Spanning Broadband Absorption of Terahertz Light Using Metasurface Fractal-Cross Absorbers. ACS Photonics.

[34] Chen D., Yang J., Huang J., Bai W., Zhang J., Zhang Z., Xu S., Xie W. (2019). The Novel Graphene Metasurfaces Based On Split-Ring Resonators for Tunable Polarization Switching and Beam Steering at Terahertz Frequencies. Carbon.

[35] Miyamaru F., Saito Y., Takeda M.W., Hou B., Liu L., Wen W., Sheng P. (2008). Terahertz Electric Response of Fractal Metamaterial Structures. Phys. Rev. B.

[36] Hanson G.W. (2008). Dyadic Green’s Functions for an Anisotropic, Non-Local Model of Biased Graphene. IEEE Trans. Antenn. Propag..

[37] Llatser I., Kremers C., Cabellos-Aparicio A., Jornet J.M., Alarcón E., Chigrin D.N. (2012). Graphene-Based Nano-Patch Antenna for Terahertz Radiation. Photonics Nanostruct. Fundam. Appl..

